# Increasing sustainable cataract services in sub-Saharan Africa: an experimental initiative

**Published:** 2014

**Authors:** Sasipriya M Karumanchi, Thulasiraj Ravilla

**Affiliations:** Senior Faculty: Lions Aravind Institute of Community Ophthalmology, Madurai, India.; Executive Director: Lions Aravind Institute of Community Ophthalmology, Aravind Eye Care System, Madurai, India

**Figure:**
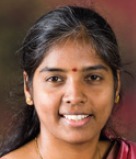
Sasipriya M Karumanchi

**Figure:**
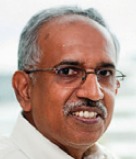
Thulasiraj Ravilla

To begin to meet the need for cataract surgery in sub-Saharan Africa, the cataract surgical rate (CSR) should be at least 2,000 to 3,000; i.e. there should be 2,000-3,000 cataract operations per million population, per year. The current levels are below 1,000 (and often much lower). Sub-Saharan Africa poses a unique set of challenges: low population density; inadequate transportation systems that inhibit access; big differences in wealth; and a shortage of eye care resources (which are usually concentrated in larger cities). Additional issues relate to productivity, the supply chain and the quality of outcomes,^1^ all of which contribute to the low cataract surgical rates. It is in this context that the Hilton Foundation sought to enhance cataract surgical services in sub-Saharan Africa, through the Hilton Cataract Initiative.

## Organisations involved in the initiative and their roles (Figure 1)

There are three international partners.

- The Hilton Foundation (provides funding)
- The Dana Center for Preventive Ophthalmology, Wilmer Eye Institute, Johns Hopkins University, USA, (supports learning)
- Lions Aravind Institute for Community Ophthalmology, Aravind, India (offers consulting and mentoring support)

**Figure 1.**
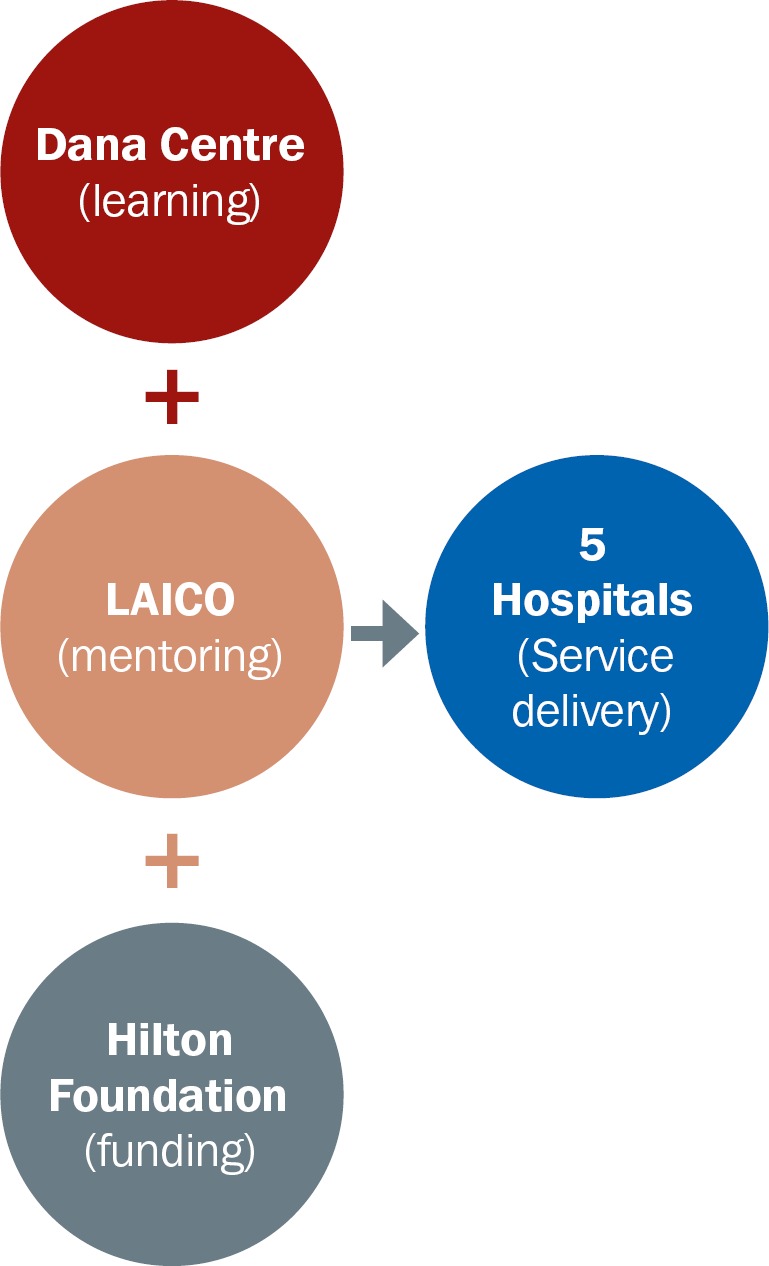
Organisations Involved in the initiative

The three partners are working with five hospitals in sub-Saharan Africa.

- **Kitwe Central Hospital, Zambia:** A public hospital, located in the Copper Belt Province.
- **Innovation Eye Centre, Kenya:** A private venture, located in Kisii, 300 km from Nairobi.
- **UHEAL Foundation, Kenya:** A Public-Private Partnership, located in Nairobi to serve the poor.
- **Deseret Community Vision Institute, Nigeria.** A for-profit institution with a not for profit arm, located near Lagos.
- **Fitsum Birhan Specialized Eye Clinic, Ethiopia:** A private venture, located in Mekele in Tigray province.

The project aspires to build the five hospitals' capacity to perform an additional 22,000 cataract operations per year with high quality outcomes. This would increase the CSR in their service areas by 1,000. While the targets were set for cataract, there is an equal emphasis on comprehensiveness in the provision of eye care and the sustainability of this outcome beyond the project period.

## Needs assessment and strategic planning

Each hospital set realistic, yet ambitious targets for the number of cataract operations they would aim to perform each year. The targets were based on the unmet need. This was calculated as the number of cataract operations needed in the service area, minus the current number of operations. and their capacity (their current capacity, together with the proposed enhancements during the project period).

Each hospital developed institution-and context-specific strategies and action plans covering the following domains.

- **Demand generation,** including attracting more patients in need of cataract surgery, with a focus on the poor and those who struggle to come to the hospital, for whatever reason.

**Figure:**
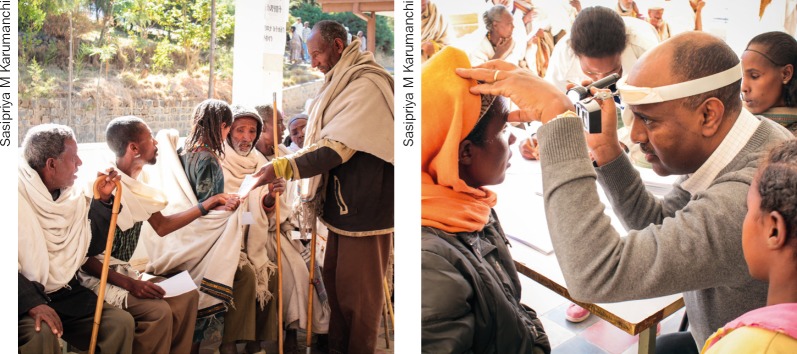
At a screening camp organised by Fitsum Berhan Eye Clinic. ETHIOPIA
- **Human resources:** both the number of people required (capacity) and the skills required (competence).
- **Quality:** measuring and ensuring high quality clinical outcomes and patient satisfaction.
- **Operating efficiency:** ensuring that scarce resources are optimally used.
- **Financial viability:** pricing to ensure affordability, and putting in place revenue and cost control strategies.

**Table 1.** Current and target annual cataract surgical output

Hospital (primary service population)	Number of operations per year		% of increase
	Current	Target	
UHEAL Foundation, Kenya (5 million)	0	5,000	New venture
Innovation Eye Centre, Kenya (5 million)	210	5,600	Just started
Fitsum Berhan Speciality Eye Centre, Ethiopia (5 million)	6,016*	10,000	282%
Eye Foundation, Nigeria (5 million, plus 15 milion reached through outreach)	1,476	5,000	239%
Eye Dept, Kitwe Central Hospital, Zambia (5 million)	2,500	6,200	180%
**Five hospitals (Population: 40 Million)**	**10,202**	**31,800**	**213%**
*3,400 of these operations were performed during surgical outreach, supported by a special one-off programme			

## What have we learned so far?

After one year of data collection and initial training, the project is in the early stages of implementation. Strategic plans at each of the hospitals are being finalised and implemented. Site visits have been made and baseline details (including organisational practices and procedures) are being documented. In July 2014 all three international partners and the five hospitals met for an initial review of developments and to share lessons.

While there are challenges specific to each of the hospitals, the following areas required attention in nearly all of the hospitals:

1. **Patient volumes.** Inadequate patient volumes mean that it is necessary to take a closer look at patient experience and develop proactive strategies for increasing patient access and demand.

**Figure:**
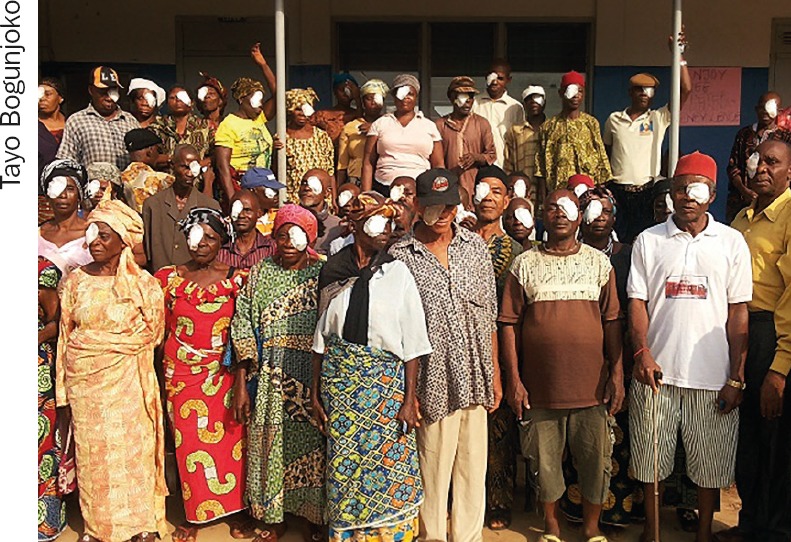
After cataract surgery at Deseret Community Vision Institute. NIGERIA
2. **Patient-centred design.** Current processes and systems are biased towards serving the interests of the hospital rather than those of the patients.
3. **Human resources.** A shortage of staff members, as well as imbalances in the composition of the teams' skills and expertise, needed to be addressed.
4. **Administration.** Better systems were needed to bring about higher efficiency in day-to-day activities.
5. **Managing with evidence.** There is inadequate generation and use of evidence for making decisions. This is due to a lack of systems for obtaining needed evidence, as well as the lack of systems for routinely using evidence to guide continuous improvement of processes.

The journey over the next few years should assist the hospitals in developing innovative and sustainable strategies for reaching their goals. The process will no doubt be iterative (i.e., will be repeated), and will benefit from shared learnings.

Universal eye health (UEH) calls for:

- An increase in access to health care with the goal of providing 100% (universal) access.
- An increase in the range of services offered, with the goal of offering fully comprehensive eye care.
- Making services affordable with the goal that no-one is excluded from eye care because of cost.

Several not-for-profit organisations have significantly contributed towards UEH in India. The *Community Eye Health Journal* looks forward to learning about the outcomes of this initiative in Africa.
